# Mineral Manganese Oxides as Oxidation Catalysts: Capabilities in the CO-PROX Reaction

**DOI:** 10.1021/acssuschemeng.1c00343

**Published:** 2021-04-26

**Authors:** Arantxa Davó-Quiñonero, Sergio López-Rodríguez, Esther Bailón-García, Dolores Lozano-Castelló, Agustín Bueno-López

**Affiliations:** Inorganic Chemistry Department, University of Alicante, Carretera San Vicente del Raspeig s/n, E-03080 Alicante, Spain

**Keywords:** rare earths, cryptomelane, cerium oxide, CO-PROX, reaction mechanism, isotopic oxygen, oxygen exchange

## Abstract

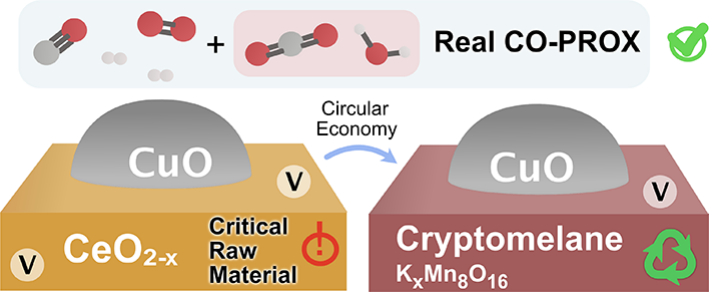

Cryptomelane is an abundant mineral manganese oxide with unique physicochemical features. This work investigates the real capabilities of cryptomelane as an oxidation catalyst. In particular, the preferential CO oxidation (CO-PROX), has been studied as a simple reaction model. When doped with copper, the cryptomelane-based material has revealed a great potential, displaying a comparable activity to the high-performance CuO/CeO_2_. Despite stability concerns that compromise the primary catalyst reusability, CuO/cryptomelane is particularly robust in the presence of CO_2_ and H_2_O, typical components of realistic CO-PROX streams. The CO-PROX reaction mechanism has been assessed by means of isotopic oxygen pulse experiments. Altogether, CuO/CeO_2_ shows a greater oxygen lability, which facilitates lattice oxygen enrolment in the CO-PROX mechanism. In the case of CuO/cryptomelane, in spite of its lower oxygen mobility, the intrinsic structural water co-assists as active oxygen species involved in CO-PROX. Thus, the presence of moisture in the reaction stream turns out to be beneficial for the stability of the cryptomelane structure, besides aiding into the active oxygen restitution in the catalyst. Overall, this study proves that CuO/cryptomelane is a promising competitor to CuO/CeO_2_ in CO-PROX technology, whose implementation can bring the CO-PROX technology and H_2_ purification processes a more sustainable nature.

## Abstract 2

The mineral cryptomelane is a green catalytic substitute to CeO_2_ in the preferential CO oxidation under realistic conditions (CO_2_ + H_2_O).

## Introduction

Rare-earth elements (REE), with manifold uses in civil, industrial, and military sectors as permanent magnets or housing and electronic components, are considered critical raw materials since potential disruptions in their supply would put our technology developments at risk.^1^ Today, the REE market is rendered to a vulnerable chain supply by the dominance of China, which holds a powerful geostrategic position.^2,3^ Besides, REE manufacture relies on polluting practices with dramatic environmental impact. Regretably, REE recycling technologies are not commercially implementable yet and merely supply 1% of the market.^4^ Therefore, it is crucial to lower the REE demand by seeking functional substitute materials. This goal is a big scientific challenge owing to their unique magnetic, electrical, and optical properties, besides their well-recognized catalytic features.^5^

For instance, cerium oxides are renowned oxidation catalysts which have widely proven near-optimal catalytic performance several applications.^6^ Among reliable catalytic alternatives to cerium oxide materials, manganese oxides are promising candidates. In contrast to REE minerals, mineral manganese oxides are abundant and their extraction and beneficiation can be achieved by means of non-toxic, inexpensive, and environmentally-friendly procedures.^7^ In terms of catalytic activity, the mineral manganese oxide with the most promising catalytic properties is cryptomelane, which has attracted attention in the last years and centered some fundamental studies.^8−10^ The versatility of cryptomelane-based materials is due to their high porosity, acidity, hydrophobicity, electronic and ionic conductivities, and easy removal of lattice oxygen and recovery. These features are provided by the facile redox cycling among Mn^2+^, Mn^3+^, and Mn^4+^ states, leading to an average manganese valence of *ca.* 3.8. Chemically, cryptomelane is a potassium–manganese mixed oxide consisting in a tunneled structure formed by double chains of corner-sharing MnO_6_ octahedra basic units. The channel size left in between the 2 × 2 octahedra arrangement is 0.46 × 0.46 nm, and K ionic species and water molecules are hosted inside providing structural stability. The structure and composition of mineral cryptomelane can be achieved easily by inexpensive laboratory procedures leading to a synthetic material commonly named as octahedral molecular sieve 2 × 2 (OMS-2).^11^

In addition, it is well-known that the redox properties of cryptomelane can be tuned by means of the introduction of different framework dopants.^12−14^ In particular, the best catalytic improvements have been achieved with Cu^2+^ doping,^15,16^ in an interesting analogy with the first CO oxidation hopcalite catalysts.^17^ On the other hand, the interfacial redox properties occurring by means of synergistic effects in Cu-doped cryptomelane display similarities of the well-reported CuO/CeO_2_ catalysts.^18^ As a result of them, although Cu-modified cryptomelane materials are known to be more catalytically active systems than bare cryptomelane, Cu doping leads to a low thermal stability, which is a challenging limitation for their durable utilization in a hypothetic implemented use.

This work is aimed to provide a critical analysis of the feasibility of Cu-doped cryptomelane in terms of activity and stability to study its potential implementation and substitution of cerium oxides. Herein, a straightforward comparison between CuO/CeO_2_ and CuO/cryptomelane catalytic systems is presented for the preferential CO oxidation reaction (CO-PROX) as the model case, which is relevant for the H_2_-rich stream purification.^19^ The catalytic performance and reusability under different gas mixtures has been tested, and the catalyst state after utilization has been carefully characterized. In addition, isotopic oxygen exchange experiments performed on the CuO/CeO_2_ and CuO/cryptomelane systems have allowed to draw key mechanistic differences on the CO-PROX mechanism for the first time. In this study, the nature of the diverse active oxygen species existing in the ceria and the cryptomelane-based catalyst has been elucidated and their contributions in the CO oxidation mechanism evaluated. The results reveal promising opportunities for CuO/cryptomelane catalysts in the CO-PROX application, especially in the presence of moisture, because H_2_O aids the regeneration of labile oxygen species in the cryptomelane structure. Thus, the implementation of cryptomelane-based catalysts is proven to be an efficient and more sustainable approach into the exhaustive catalytic CO clean-up required for H_2_ safe use.

## Experimental Section

### Catalyst Synthesis

Two CuO/CeO_2_ and CuO/cryptomelane catalysts were prepared with equivalent Cu nominal target on each metal oxide support. CeO_2_ support was obtained via thermal decomposition of cerium(III) nitrate hexahydrate (Panreac) following a “flash” calcination at 500 °C in a preheated muffle furnace at 200 °C.^20 ,21^ Synthetic cryptomelane was prepared following an adaptation from the so-called reflux method, procedure described by DeGuzman et al.^22,23^ Particularly, 11 g of manganese(II) acetate (Aldrich) were dissolved in 40 g of water in a solution with a pH fixed at 5. After 30 min of reflux heating, potassium permanganate (Aldrich) solution (6.5 g/100 mL) was introduced and the boiling mixture was maintained with vigorous stirring for 24 h. The resulting dark-colored substance was filtered, washed until neutral pH, and dried at 120 °C overnight; its calcination at 450 °C for 2 h led to cryptomelane.

Once supports were prepared, the grinded powders were impregnated with an aqueous solution of Cu(NO_3_)_2_·(5·1/2)H_2_O (Panreac) to a target 5% nominal w. Cu content following the incipient wetness impregnation methodology. The impregnated samples were calcined to obtain CuO/CeO_2_ and CuO/cryptomelane catalysts following the same heating protocol as the respective supports.

### CO-PROX Activity Tests

The prepared materials were tested in CO-PROX catalytic activity experiments using a regular 100 mL/min flow of the gas reactant mixture (2% CO, 2% O_2_, 30% H_2_), set by means of Mass Flow Controllers (Bronkhorst). 150 mg of catalyst was placed in a quartz fixed-bed reactor (16 mm inner diameter, GHSV = ∼30,000 h^–1^) following a slow-pace heating ramp of 2 °C/min up to 200 °C. The reaction progress was monitored with a gas chromatograph HP model 6890 Plus Series coupled to a thermal conductivity detector. The effect of (1) CO_2_, (2) H_2_O and (3) CO_2_ + H_2_O in the catalytic activity was studied by means of the introduction of 9% CO_2_, 5% H_2_O, and 9% CO_2_ + 5% H_2_O, respectively, in the reactant gas mixture feeding.

### Catalyst Characterization

N_2_ adsorption–desorption isotherms were performed in an automatic volumetric system (Autosorb-6, Quantachrome) after outgassing the samples at 150 °C for 4 h (Figure S1, Table S1). Transmission electron microscopy (TEM) characterization was conducted using a JEOL (JEM-2010) microscope (Figure S2). Fresh and spent samples were characterized by means of X-ray diffraction (XRD) for the crystalline resolution using a Bruker D8-ADVANCE diffractometer using Cu Kα radiation. Diffractograms were recorded at 2θ between 10 and 90°, with a step size of 0.05° and a time of 3 s per step.

Temperature-programmed reduction experiments with H_2_ (H_2_-TPR) were conducted in a Micromeritics Pulse Chemisorb 2705 instrument. 40 mg of the catalyst was placed in a quartz tubular reactor under 40 mL/min of 5% H_2_/Ar gas mixture following a heating ramp of 10 °C/min.

Temperature-programmed desorption (TPD) experiments were conducted with 80 mg of the catalyst after a pre-treatment at 400 °C for 30 min in a 100 mL/min flow of Ar. Then, a saturation step with the selected gases was carried out, which consisted of heating the catalyst at 150 °C for 1 h under 100 mL/min of 10% CO_2_/Ar (for CO_2_-TPD), 5% H_2_O/Ar (for H_2_O-TPD), or 10% CO_2_ + 5% H_2_O/Ar (for CO_2_ + H_2_O-TPD). After that, the gas mixture was switched to Ar, and once CO_2_ and H_2_O signals were stabilized, the reactor was heated from 150 to 650 °C following a ramp of 10 °C/min in 100 mL/min of Ar.

### Isotopic ^36^O_2_ Pulse Experiments

Isotopic exchange experiments were carried out with ^36^O_2_ by means of an injection valve with a loop (100 μL) and two high sensitivity pressure transducers. The experiments were carried out in a fixed-bed tubular quartz reactor with 80 mg of catalyst in a constant 20 mL/min of 1% CO, 30% H_2_-balanced He feeding mixture. The exhaust gases were monitored with MS, and the ^36^O_2_ pulses (Isotec, 99%; 100 μL and 9 psi) were injected at 75, 100, and 150 °C once achieving signal stabilization. Prior to this, several pulses of Ar (100 μL and 9 psi) were used as a test to confirm reproducibility of the method.

## Results

### CO-PROX Activity Tests

**Figure 1:**
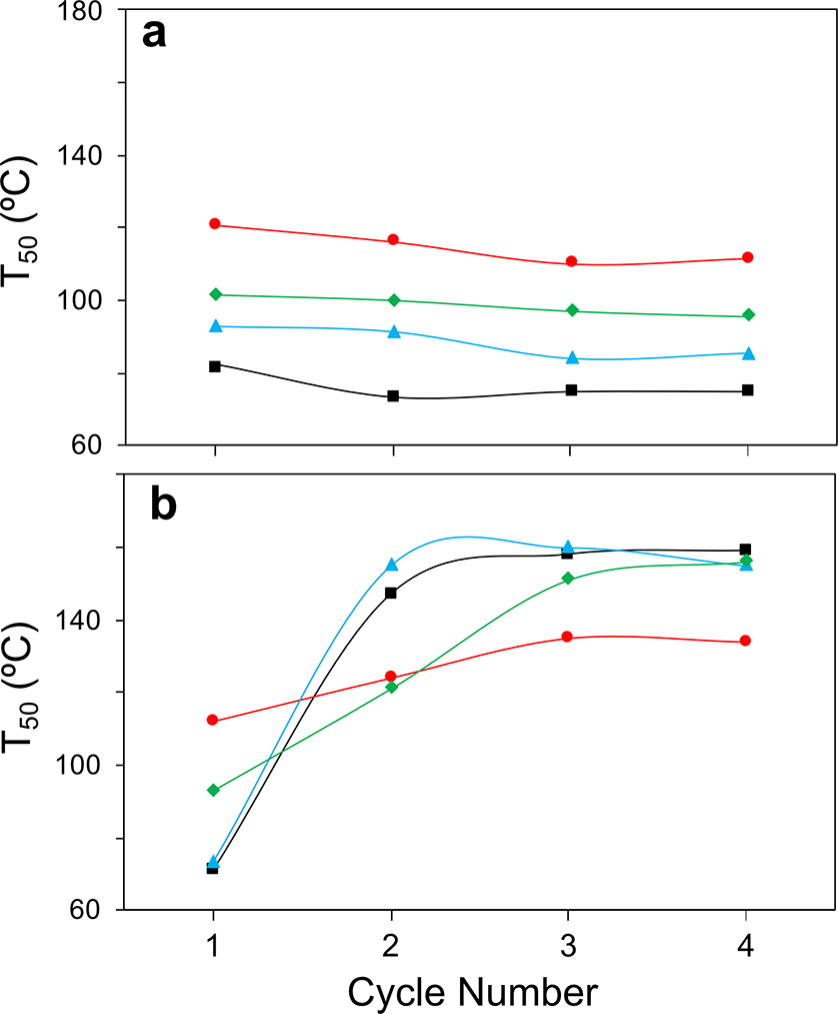
Temperature for the 50% of CO conversion (*T*_50_) in 1–4 cycles of CO-PROX reaction in different environment mixtures: CO + O_2_ + H_2_ (squares), CO + O_2_ + H_2_ + CO_2_ (triangles), CO + O_2_ + H_2_ + H_2_O (diamonds), CO + O_2_ + H_2_ + CO_2_ + H_2_O (circles) for (a) CuO/CeO_2_; (b) CuO/cryptomelane catalysts.

Figure 1a,b shows the temperature for the 50% of CO conversion (*T*_50_) along four consecutive CO-PROX catalytic runs, which allows us to evaluate the stability and recyclability of CuO/CeO_2_ and CuO/cryptomelane catalysts. The CO-PROX light-off curves are compiled in Figure S3a,b,c,d, respectively. As shown, both catalysts reach CO conversion (and CuO/Cryptomelane catalysts are compiled in Figure S3a,b and Figure S3c,d, respectively). As shown, both catalysts reach CO conversion (*X*_CO_) values of 94–98% in the low temperature window of CO-PROX regardless the conditions, besides CO selectivity (*Sel.*) is maintained close to the optimum (50% according to the feeding O_2_/CO stoichiometry).

The inhibiting effect of the CO_2_, H_2_O, and CO_2_ + H_2_O mixture in the reactor feeding stream follows the trend CO_2_ < H_2_O < CO_2_ + H_2_O for both catalysts, in agreement with the reported literature.^15,24−26^ Interestingly, the impact of H_2_O in the CO oxidation catalytic activity results more detrimental than CO_2_ itself. In CuO/CeO_2_, the interaction with CO_2_ leads to an intense carbonatation besides the formation of stable formates, carboxylates, and bicarbonates, which hamper Cu–Ce interaction and inhibit CO oxidation.^24,25^ On the contrary, the CO-PROX catalytic activity of CuO/cryptomelane is fairly not affected by CO_2_, but it is very sensitive to H_2_O. On the other hand, CO_2_ + H_2_O conditions lead to the strongest inhibition state, suggesting the participation of both species in co-adsorptive processes.^15,27^

According to Figure 1, CuO/CeO_2_ presents a stable behavior within four cycles of reaction in all the tested conditions, while CuO/cryptomelane suffers from a deactivating process of different magnitude as seen by the *T*_50_ increase alongside the number of reaction runs. This degradation is spurred by ongoing oxidation–reduction cycles during CO-PROX that lead to the inactive Mn_3_O_4_,^8,28^ which was proven to be partially reversible by means of oxidative regeneration treatments.^29^

Comparing both catalysts, CuO/cryptomelane presents an enhanced catalytic activity with regard to CuO/CeO_2_ in a first run for all the set of conditions. However, the deactivation of CuO/cryptomelane leads to the opposite trend beyond the second cycle. CO_2_ + H_2_O conditions confer the greatest stability to CuO/cryptomelane and in result, the best performance in the fourth cycle. In this atmosphere, CuO/cryptomelane displays an admirable activity as compared with CuO/CeO_2_ (*T*_50_ of 134 and 117 °C, respectively). Because CO-PROX reaction under CO_2_ and H_2_O presence is the most challenging, but also the most representative of the realistic operation, the promising potential of CuO/cryptomelane deserves to be further investigated.

### Understanding the Catalyst Stability under CO_2_ and H_2_O Mixtures

As typical for Mn^4+^-based minerals, cryptomelane is dark-colored and operando infrared spectroscopy measures fail due to its high absorption, showing black-out IR spectra. Therefore, the study of the reaction mechanism must be assessed by alternative and complementary techniques.

The Supporting Information document contains the characterization results of fresh and spent catalyst samples in the different conditions tested. Namely, XRD (Figure S4a,b, Tables S2 and S3) and H_2_-TPR (Figure S5a,b) which confirm that CuO/CeO_2_ catalyst presents a robust crystalline structure, whereas CuO/cryptomelane displays a poor stability under CO-PROX reaction in CO + O_2_ + H_2_ conditions, after which, Mn_3_O_4_ (hausmannite) is the main crystalline phase. The transition from cryptomelane (K_x_Mn_8_O_16_) to Mn_3_O_4_ involves the collapse of the characteristic 2 × 2 tunnels of cryptomelane besides the reduction of Mn cations from an average oxidation state of *ca.* +3.8 to +2.5. The degradation of the cryptomelane microstructure occurs when the intratunnel K is segregated to the outer surface and probably released in the form of K volatile species.^30,31^ The loss of charge-compensating K species leads to the reduction of the manganese ions left upon cryptomelane collapse.^32,33^Figure S2e,f shows TEM images of the deactivated CuO/cryptomelane catalyst, displaying non-aggregated Mn oxide particles around the deteriorated nanorod array. In our previous work,^29^ we studied the CuO/cryptomelane deactivated material left upon CO-PROX cycles, and no significant textural differences compared to the fresh sample related to this transition phase were found. However, we reported evidences of potassium segregation, manganese reduction, and the formation of copper species with high charge density. The presence of these partially reduced copper species with the atypical XPS binding energy of *ca.* 930.5 eV is well reported for the hopcalite CuMn_2_O_4_ spent and deactivated material, which suffers an amorphous to crystalline transition.^34,35^ In the case of deactivated CuO/cryptomelane samples, Cu^*n*+^ species at that low binding energy are ascribed to Cu^+^ being located in an octahedral site in the spinel structure, subjected to a larger extra-atomic relaxation energy.^17,36^ Finally, as in the case of the CuO/CeO_2_ catalyst, tenorite peaks from the segregated CuO phase is not detectable in the X-ray diffractograms of CuO/cryptomelane (see Figure S4a,b). The absence of CuO peaks reveals that the copper phase is well dispersed before and after the reaction runs. On the other hand, the co-addition of CO_2_ + H_2_O in the CO-PROX gas reactant mixture leads to the preservation of the cryptomelane structure (see Table S3).

Regarding the redox features, H_2_-TPR profiles (Figure S5a,b) reveal that CuO/CeO_2_ samples experience changes in Cu–Ce interaction after the CO-PROX reaction cycles, which are hints of CuO sintering or Cu–Ce contact modification.^37,38^ However, these do not reflect into any sort of activity loss. For CuO/cryptomelane, H_2_-TPR profiles exhibit large differences depending on the catalyst state. Nevertheless, the spent sample in CO_2_ + H_2_O conditions shows discrepancies with the fresh sample; the manganese reduction events roughly keep the shape and position in the profile. For the sample used in the experiments free of CO_2_ + H_2_O, the reduction profile presents a very different aspect, which can be attributed to the cryptomelane phase distortion and reduction toward the hausmannite phase.

**Figure 2:**
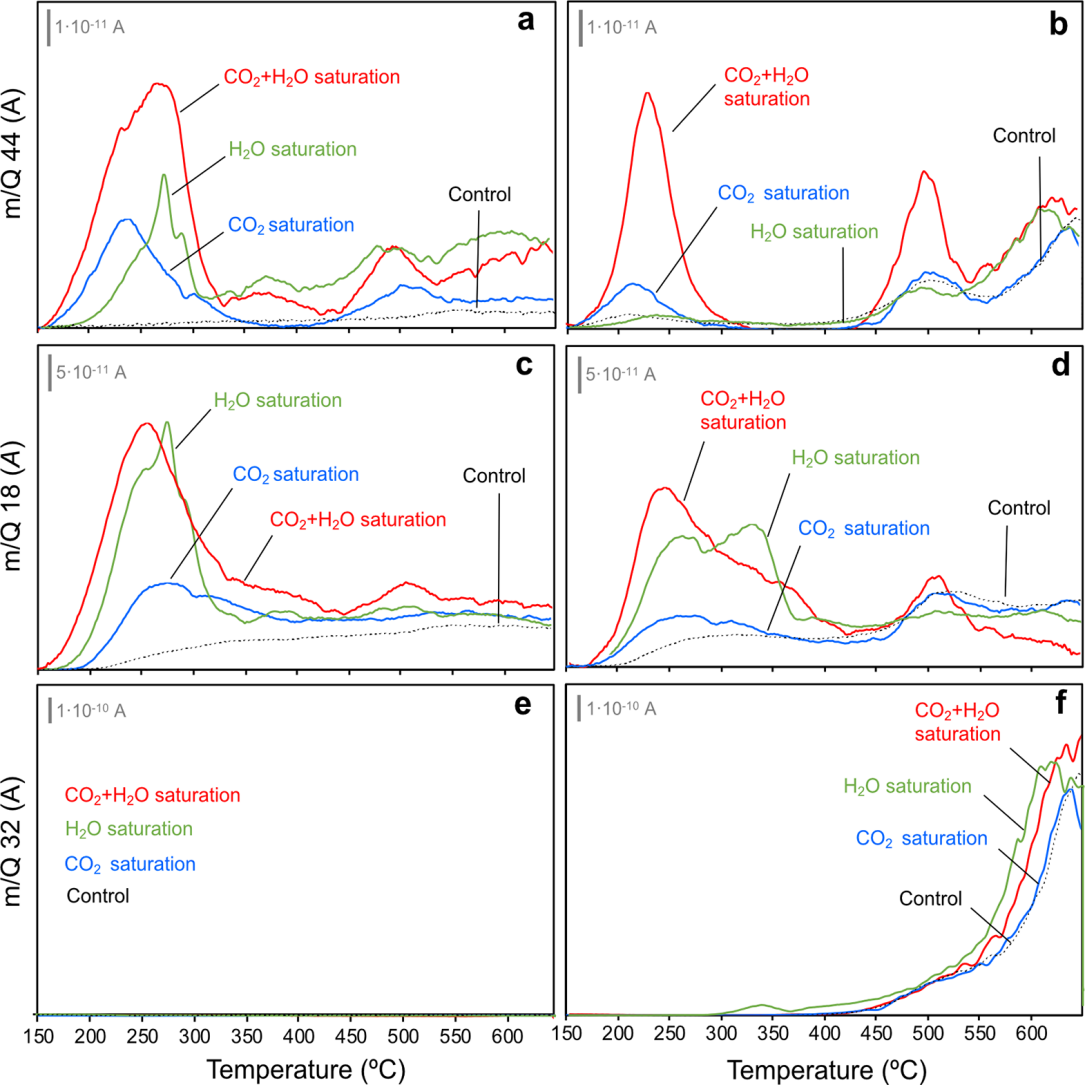
Monitored MS signals (a,b) 44, CO_2_; (c,d) 18, H_2_O; and (e,f) 32, O_2_ for (a,c,e) CuO/CeO_2_; and (b,d,f) CuO/cryptomelane catalysts in CO_2_-TPD; H_2_O-TPD; and (CO_2_ + H_2_O)-TPD experiments.

TPD experiments (Figure 2a–f) for CuO/CeO_2_ and CuO/cryptomelane after CO_2_, H_2_O, and CO_2_ + H_2_O exposures provide relevant insights about the catalyst interaction with CO_2_ and H_2_O. As depicted, CeO_2_ has a great capacity to stabilize carbonaceous species on surface due to its high basicity. On the contrary, cryptomelane has very moderate affinity for CO_2_, in agreement with the low impact of CO_2_ addition in the CO-PROX activity tests. In both cases, H_2_O is co-released, which can be attributed to the depletion of the inherently present hydroxyls upon carbonate and bicarbonate decomposition. Analogously, after H_2_O saturation, H_2_O and CO_2_ co-evolve and CO_2_–H_2_O interaction is maximized, as CuO/CeO_2_ exhibits in these conditions a larger CO_2_ release than after CO_2_ saturation itself.^39^ Thus, H_2_O in contact to CuO/CeO_2_ catalyst may favor the release of intrinsically present stable carbonates.

In the case of CuO/cryptomelane, the CO_2_–H_2_O co-release upon the individual gas exposure is not so relevant, and on the other hand, cryptomelane is much more prone to interact with H_2_O than with CO_2_. However, the concomitant addition of CO_2_ + H_2_O leads to a sharp growth of CO_2_ and H_2_O release, which is indicative of a favored CO_2_–H_2_O co-adsorption as in the case of CuO/CeO_2_. For CuO/cryptomelane, since it shows a low affinity toward bare CO_2_ chemisorption, CO_2_ is inferred to be retained as hydrogenated carbon intermediates, such as bicarbonates on the cryptomelane acidic surface.

Finally, the O_2_ profile (MS signal of 32, Figure 2e) shows that CuO/CeO_2_ is thermally stable since it presents a negligible flat product release up to the maximum temperature of the experiment (*i.e.*, 650 °C). In contrast, CuO/cryptomelane displays a significant O_2_ release (Figure 2f) which starts decomposing above 450 °C. Remarkably, the CuO/cryptomelane decomposition occurs equally regardless the nature of the saturation treatment, which differs from the enhanced stability assessed for CuO/cryptomelane in the CO_2_ + H_2_O CO-PROX tests. Thus, the positive effect of CO_2_ + H_2_O on cryptomelane during the CO-PROX reaction cannot be merely superficial, and other factors must be at play involving the catalyst oxidation−reduction cyclability.

### Isotopic ^36^O_2_ Pulse Experiments

**Figure 3:**
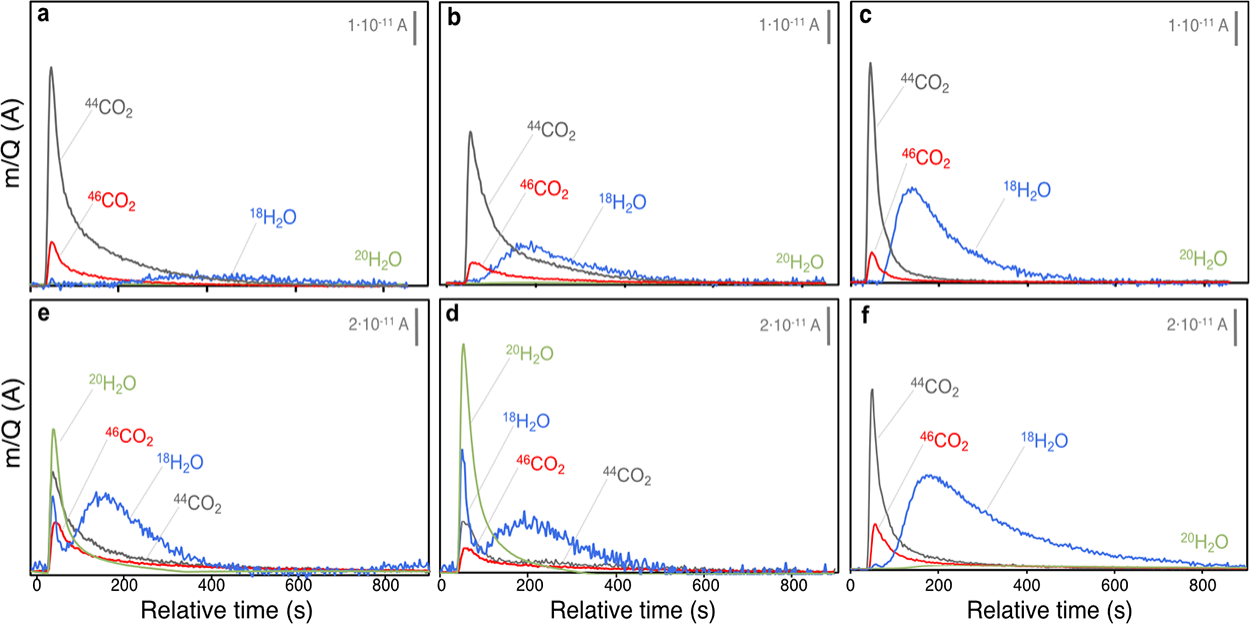
MS signals after ^36^O_2_ pulses in CO-PROX conditions with (a–c) CuO/CeO_2_ and (d–f) CuO/cryptomelane catalyst at (a,d) 75, (b,e) 100, and (c,f) 150 °C. Zero-time refers to the pulse injection in the reactor.

**Figure 4:**
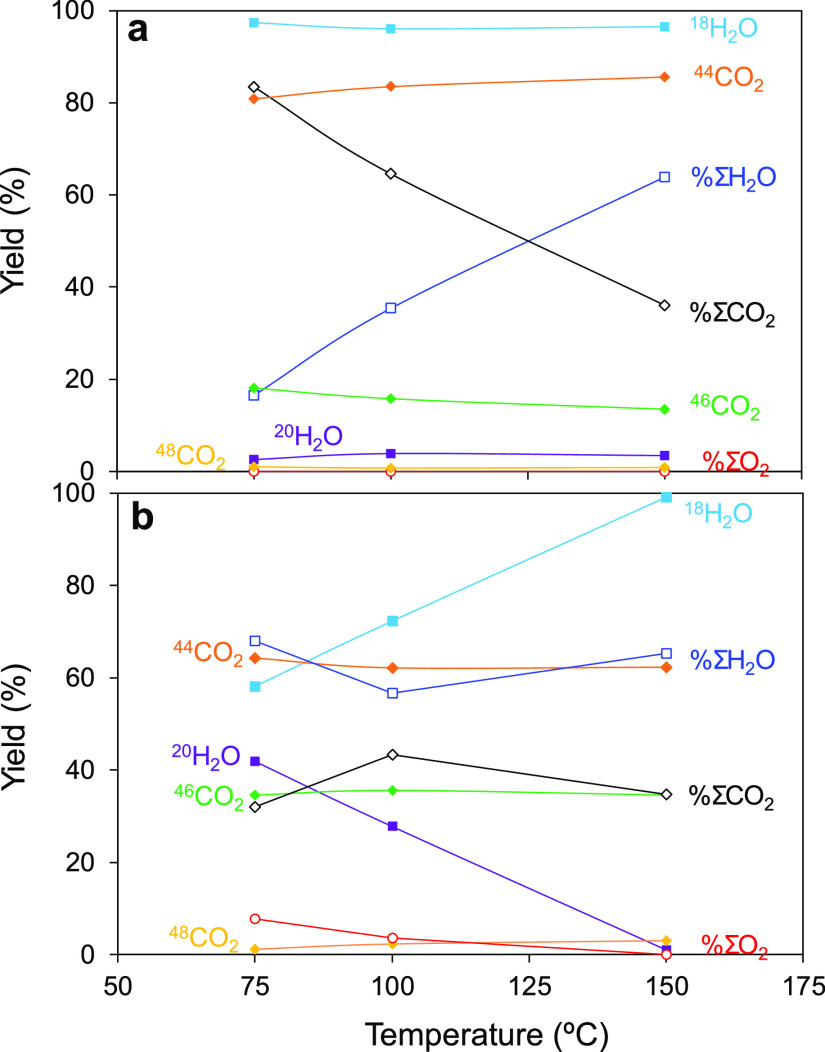
MS-normalized signals after ^36^O_2_ pulses in CO-PROX conditions with (a) CuO/CeO_2_, (b) CuO/cryptomelane catalyst at 75, 100, and 150 °C. (Solid symbols): overall O_2_ released species in the outlet flow; (open symbols): isotopic distribution among H_2_O and CO_2_ species.

Pulse oxygen isotopic experiments in CO-PROX conditions at selected temperatures were conducted for both CuO/CeO_2_ and CuO/cryptomelane samples (Figure 3a–f). For the critical comparison between both catalysts, the monitored MS signals were normalized in terms of total O species (CO_2_ + H_2_O + O_2_). The time evolution of the pulses at different temperatures is depicted in Figure 3a–f, while the quantification of the released products is presented in Figure 4a,b. Noticeably, in the CuO/CeO_2_ catalyst, no trace of O_2_ signals was found at any of the tested temperatures after the ^36^O_2_ pulse. This indicates that the catalyst fully uptakes the incoming O_2_, accommodating oxygen into the lattice as the anionic vacancies created upon the reducing conditions of the experiment are refilled. This labile restorage after the isotopic oxygen pulse leads to the destabilization of the adsorbed CO and H_2_ molecules, being released as and their oxidation products (*i.e.*, CO_2_ and H_2_O). The analysis of the product distribution evolved reveals that CO and H_2_ oxidation reactions occur mainly involving lattice oxygen (^16^O), as well reported for CuO/CeO_2_ catalysts displaying a Mars–van Krevelen (MVK).^40−42^ In this regard, the temperature does not affect significantly the isotopic product share within the 75–150 °C range, but it does to the global yield to CO_2_ and H_2_O, in good correlation with the selectivity fall observed in fixed-bed catalytic. Thus, ^20^H_2_O is not detected, whereas the release of ^18^H_2_O is delayed in time compared to CO_2_-type products. This decoupling of the CO_2_ and H_2_O evolution can be attributed to greater desorption limitations of H_2_O on the surface of CeO_2_.

For the CuO/cryptomelane catalyst, Figure 3d–f shows the evolved products after the ^36^O_2_ pulses at the different temperatures, and significant differences are found when compared to CuO/CeO_2_. First, the release of a large amount of H_2_O at low temperatures disrupts the well-correlated CO selectivity profiles of pulse experiments to fix-bed catalytic tests as in the CuO/CeO_2_ catalyst. Furthermore, the distribution of CO_2_ and H_2_O products is a mixture between isotopic and non-isotopic, rather than clearly non-isotopic as for CuO/CeO_2_, which indicates a poorer oxygen exchange capacity for CuO/cryptomelane. In order to evaluate this, Figure 4a,b shows for both catalysts the integrated signals for the released products from H_2_O and CO_2_ formation upon H_2_ and CO oxidation after the pulse (solid symbols), as well as the overall outlet H_2_O, CO_2_, and O_2_ measured (open symbols).

As depicted, CO_2_ and H_2_O profiles are more complex in the case of CuO/cryptomelane and the effect of temperature is more relevant in the distribution of the evolved species. In this case, upon interaction with the CuO/cryptomelane surface, most of the incoming isotopic ^36^O_2_ molecules leads to ^20^H_2_O, which is released after the pulse with no apparent desorption limitations. On the other hand, the oxygen pulse destabilizes the intrinsically present H_2_O molecules, resulting in a large ^18^H_2_O (non-isotopic) co-emission. In contrast with ^20^H_2_O, two different contributions of ^18^H_2_O are discerned, being one anticipated and released at the same time of ^20^H_2_O and CO_2_ species, and the other retarded as H_2_O in the CuO/CeO_2_ catalyst. According to the H_2_O desorption profile (Figure 2e), CuO/cryptomelane would release two types of H_2_O in the low-temperature region, attributed to surface-related water and water bounded inside the 2 × 2 tunnels. Hence, the first H_2_O contribution must be tentatively assigned to the mobilization of the intrinsic intratunnel water molecules that undergo a labile exchange with the incoming ^36^O_2_ molecules, resulting in a sharp co-emission of ^18^H_2_O and ^20^H_2_O. As it has been discussed elsewhere,^31,33^ the activity of the CuO/cryptomelane catalyst is related to the presence of highly mobile water species hosted in the tunnels, which provide good ionic mobility and stabilize the cryptomelane framework. Thus, the CuO/cryptomelane crystalline nature and catalytic activity features rely on such bounded H_2_O molecules, as partial O exchange in water is indeed observed. The temperature has an effect in lowering the evolution of the anticipated H_2_O, which is in agreement with the cryptomelane collapse toward the formation of the average reduced Mn_3_O_4_ spinel phase, as a result of the massive water loss caused by the CO-PROX reaction conditions. So that, at high temperatures in the CO + H_2_ conditions of the pulse experiments, the CuO/cryptomelane catalyst is gradually reduced and consequently, the amount of released water decreases, until at 150 °C, cryptomelane is in overall reduced. In such state, intra-tunnel water species are not found to be released either because they are totally depleted or because the remaining species is so tightly bounded that are not mobilized after the pulse. Thus, only beyond 150 °C, the outlet water comes solely from H_2_ oxidation reaction, and the release of such delayed H_2_O evidently has a more relevant contribution in the overall CO_2_ + H_2_O distribution than in the equivalent conditions for CuO/CeO_2_. This apparent lower CO selectivity of CuO/cryptomelane needs to be rationalized, given the large affinity of CuO/cryptomelane to water and the strong driving force found that releases intratunnel hosted species in the conditions of the experiment. On the contrary, the isotopic CO_2_ components are free of this masking effect from cryptomelane intrinsic water. Thus, compared to CuO/CeO_2_, CuO/cryptomelane exhibits a lowered contribution for the non-isotopic ^44^CO_2_ species, that is, lesser participation in the MVK mechanism due to a less active lattice oxygen species. As a result, whereas CuO/CeO_2_ efficiently uptakes the pulsed O_2_ molecules in the anionic lattice vacancies, CuO/cryptomelane exchanges O from structural water, which is partially released as isotopic ^20^H_2_O without accommodating the integrity of the O_2_ pulse. In this case, the O restorage from the pulse is high, but not complete at 75 and 100 °C (see Figure S6) since the O-catalyst preferential interaction occurs within the intratunnel water molecules rather than through direct O-lattice restoration in the MnO_6_ octahedra framework. Finally, at 150 °C, the system reaches the non-selective regime, where producing H_2_O is released as the major reaction product. Onwards, H_2_O products are majority regardless of the temperature.

## General Discussion

The relative participation of lattice oxygen in the CO oxidation (MVK) mechanism of reaction is greater in the CuO/CeO_2_ catalyst than in the CuO/cryptomelane, evidenced by the larger emission of non-isotopic products after the pulses. In contact with CuO/cryptomelane, incoming O_2_ molecules preferentially interact with the labile H_2_O species hosted in the tunnels, which are a key element of cryptomelane good oxidation activity. These species turn into quick oxygen exchange sites and mediate into the oxygen restoration, besides preventing extensive H_2_O release toward cryptomelane collapse. When H_2_O is supplied in the CO-PROX reactor, CuO/cryptomelane maintains a better performance along catalytic cycles, given its faster reoxidation capacity mediated via H_2_O. The positive complementary effect of CO_2_ cannot be ruled out since the best performance at the fourth cycle occurs in CO_2_ + H_2_O conditions, as shown in Figure 1b. A possible explanation is the maximization of the H_2_O retention capacity by the maximization of H_2_O interaction with the cryptomelane surface promoted when H_2_O and CO_2_ are co-supplied, in agreement with TPD results. TPD profiles also show that CO_2_ + H_2_O saturation incentivizes the low-temperature water desorption (150–300 °C), attributed to surface water, in detriment of the higher temperature contribution (300–375 °C), attributed to intratunnel water. Hence, CO_2_ presence aids to stabilize intratunnel water, preventing its release after CO_2_ + H_2_O contact when compared to lone H_2_O. Cryptomelane stability is improved. The characteristic redox features are better maintained with the co-addition of CO_2_, as well as CO_2_ retention is enlarged in the stabilization of hydrogen-carbonated intermediates. In turn, the surface coverage of labile carbonaceous intermediates with a facile desorption protects the O-lattice abstraction and cryptomelane reduction upon CO and H_2_ oxidation reactions, resulting in a hindered activity, but eventually a greater stability.

## Conclusions

The CO-PROX catalytic performance and reaction mechanism have been addressed in CuO/CeO_2_ and CuO/cryptomelane catalysts. In the first run, the catalytic activities of CuO/CeO_2_ and CuO/cryptomelane are comparable, being both excellent materials for this application even in real operation conditions, including CO_2_ + H_2_O in the feeding stream. In CuO/CeO_2_, both CO_2_ and H_2_O are inhibited by surface blockage, where H_2_O has more impact. On the contrary, CuO/cryptomelane is not affected by CO_2_ presence but strongly inhibited by H_2_O. In terms of cyclability and reusability, CuO/CeO_2_ maintains the activity along, at least, four catalytic cycles regardless of the ambient conditions. However, CuO/cryptomelane suffers from severe deactivation related to structural collapse and partial reduction from cryptomelane phase (MnO_2_) to hausmannite (Mn_3_O_4_), and the extent of such deactivation depends on the inlet gas mixture. Namely, CO_2_ + H_2_O conditions prevent CuO/cryptomelane decomposition, enabling to achieve the best catalytic performance at the fourth cycle, conditions where the near-optimal CuO/CeO_2_ catalyst exhibits its worst catalytic behavior.

In conclusion, CuO/cryptomelane demonstrates to be a potential competitor to CuO/CeO_2_ in CO-PROX technologies under realistic operation conditions. This outcome opens up an era of possibilities toward a sustainable non-REE based catalysis yet to scale and test in the future. Up to now, green and efficient catalysts based on active copper–manganese formulation designed in this study are proven to be sufficiently good candidates, once established the best reaction protocols. Future studies will allow improvement on the stability in long term and cyclic operations of cryptomelane-based systems, as well as to broaden the battery of active materials of similar nature. This knowledge can be extended to analogue studies of other minerals toward the design of the optimum Cu–Mn catalytic synergism.

## Supporting Information Available

Physicochemical characterization of the catalysts: N_2_ adsorption at −196 °C, transmission electron images, as well as CO-PROX catalytic activity results, XRD and H_2_-TPR results from fresh and spent samples, and additional material from isotopic oxygen pulse experiments (PDF)
