# Supplementary material for: Mineral Manganese Oxides as Oxidation Catalysts: Capabilities in the CO-PROX Reaction
Source: ACS Sustain Chem Eng. 2021 Apr 26;9(18):6329–36. doi: 10.1021/acssuschemeng.1c00343 (PMC8461565; doi:10.1021/acssuschemeng.1c00343)
Supplement: Supplementary file 1 — sc1c00343_si_001.pdf [file sc1c00343_si_001.pdf]

Mineral Manganese Oxides as Oxidation Catalysts: Capabilities in the CO-PROX Reaction

Arantxa Davó-Quiñonero[‡,†]*, Sergio López-Rodríguez[‡], Esther Bailón-García[‡], Dolores Lozano-Castelló[‡] and Agustín Bueno-López[‡]

*‡Inorganic Chemistry Department, University of Alicante, Carretera San Vicente del Raspeig s/n E-03080, Alicante, Spain. *E-mail: arantxa.davo@tcd.ie. Corresponding author.*

†Present address: School of Chemistry, CRANN and AMBER Research Centres, Trinity College Dublin, College Green, Dublin 2, Dublin, Ireland.

TABLE OF CONTENTS

N_2 physisorption isotherms......S3

Figure S1. ......S3

Table S1......S3

Transmission Electron Microscopy......S4

Figure S2. ......S4

CO-PROX Catalytic Activity Tests......S5

Figure S3. ......S5

X-Ray Diffraction......S6

Figure S4. ......S6

Table S2......S7

Table S3..S8

Temperature Programmed Reduction with H_2 (H_2-TPR)..S9

Figure S5. ....S9

Isotopic $^{36}O_2$ Pulse Experiments S10

Figure S6. ....S10

N_2 physisorption isotherms

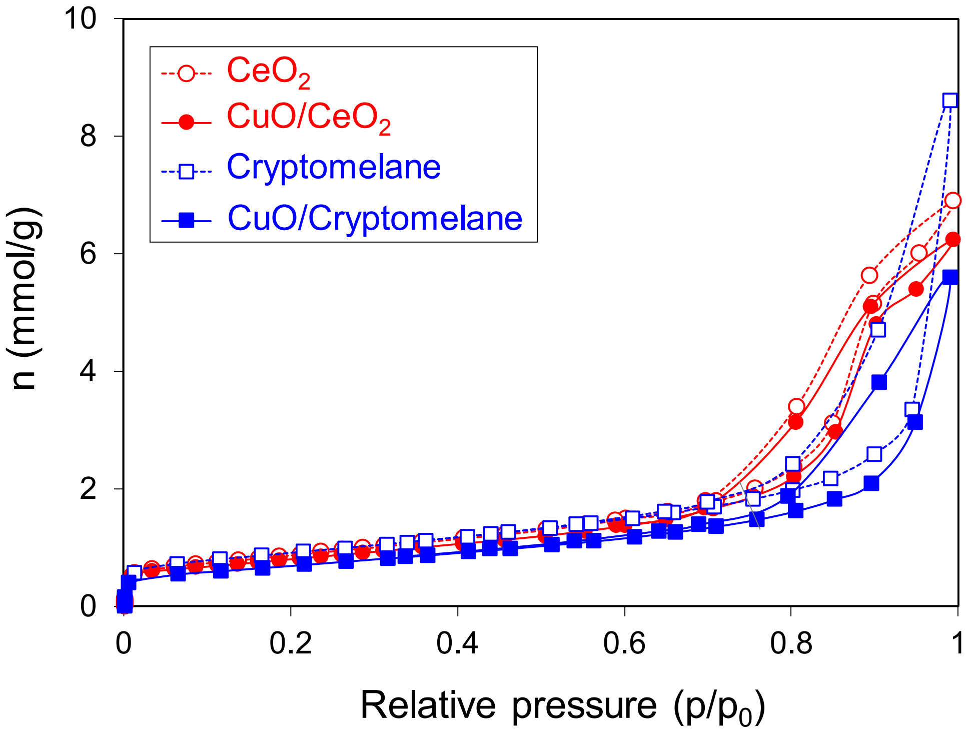

Figure S1. N_2 physisorption isotherms (–196ºC) of CuO/CeO_2 and CuO/Cryptomelane catalysts (solid symbols) and corresponding supports (open symbols).

N_2 physisorption analysis reveals show a type IV isotherm for the CeO_2 and Cryptomelane-based samples , feature of micro-/mesoporous materials. On the one hand, CeO_2 and CuO/CeO_2 exhibit a H2(b) hysteresis loop which evidences a broad pore size distribution. The lack of a plateau in the last points of adsorption branch suggests the presence of macroporosity. The presence of CuO causes surface blockage and a macroporosity loss, as evidenced by the decrease of BET surface in CuO/CeO_2. On the other hand, Cryptomelane-based materials show a H3 hysteresis, being this a typical feature of plate-like particles with slit-shaped pores.

Table S1. Textural results from N_2 physisorption analyses.

Sample	S_{BET} (m^2/g)	Pore V (cm^3/g)	Micro-pore $V_{(DR)}$ (cm^3/g)
CeO_2	71	0.24	0.04
CuO/CeO_2	65	0.22	0.04
Cryptomelane	72	0.30	0.04
CuO/Cryptomelane	56	0.19	0.03

46 **Transmission Electron Microscopy**

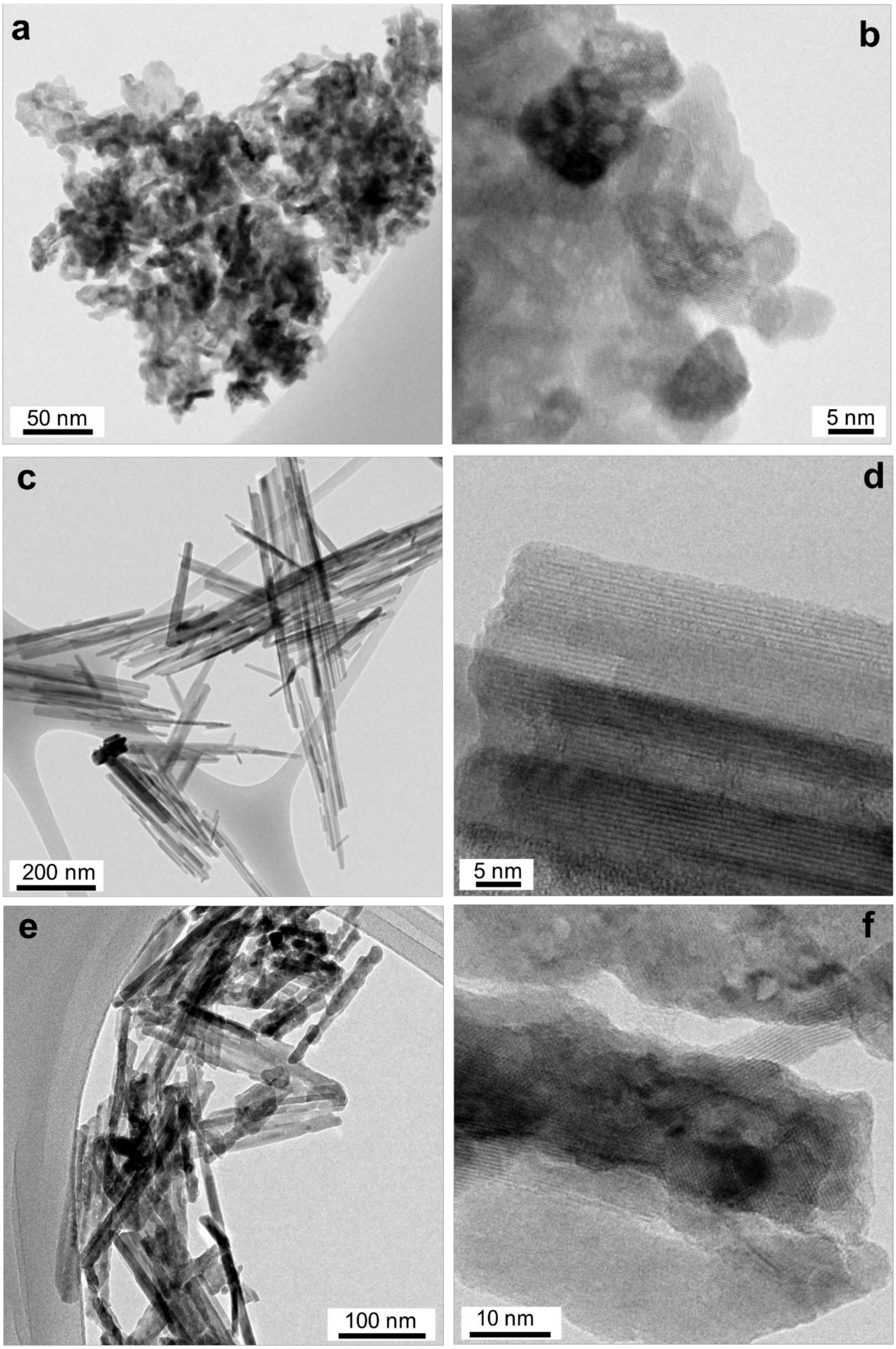

47

Figure S2. TEM images of (a,b) fresh CuO/CeO_2, (c,d) fresh CuO/Cryptomelane materials (e,f) deactivated CuO/Cryptomelane catalyst.

Figures S2a and S2b display transmission electron microscopy images of the fresh polycrystalline CuO/CeO_2 sample, exhibiting an irregular particle size in the range 10–20 nm. The low contrast between Cu and Ce-rich phases does not allow to discern CuO disperse particles. Figure S2c and S2d show TEM images of fresh CuO/Cryptomelane material where the characteristic nanorod organization of cryptomelane microstructure is exposed. As in the case of the CuO/CeO_2 catalyst, the CuO particles are not discernible due to the low Cu–Mn contrast.

Finally, Figures S2e and S2f display the microstructure of the CuO/Cryptomelane catalyst in its deactivated form after 4 CO-PROX cycles tests ($CO + O_2 + H_2$ conditions).

CO-PROX Catalytic Activity Tests

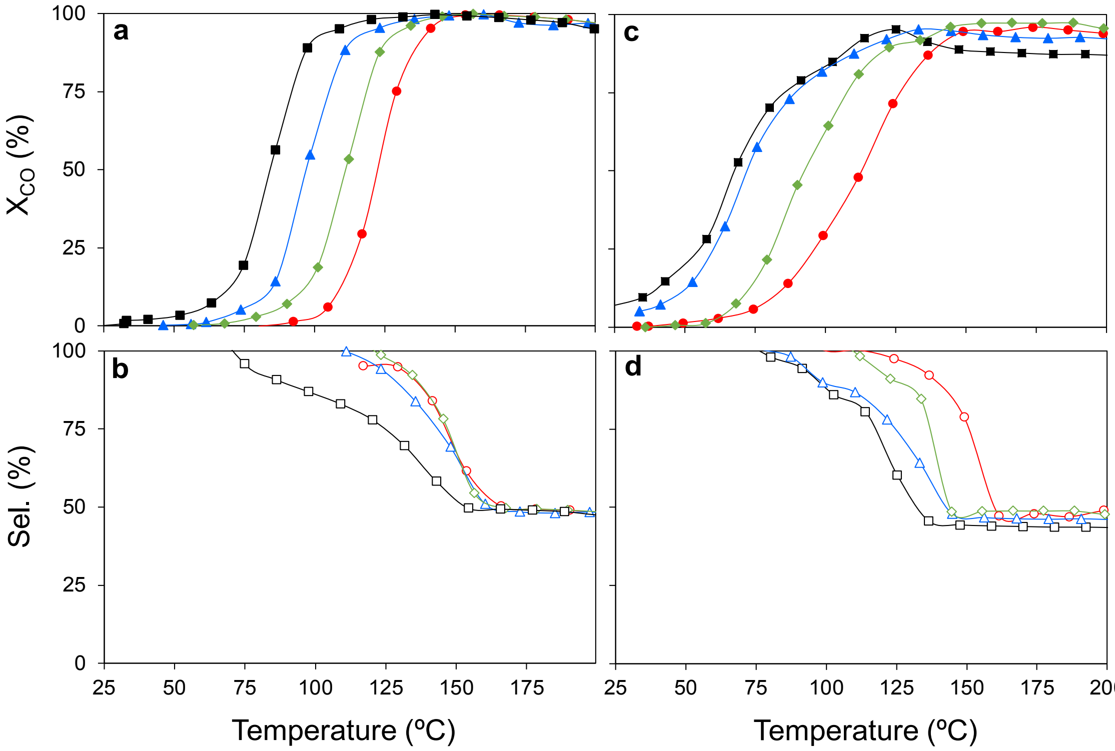

Figure S3. CO-PROX activity results in terms of (a,c) CO conversion (X_{CO}, %); and (b,d) CO selectivity (*Sel.,* %) with (a,b) CuO/CeO_2; and (c,d) CuO/Cryptomelane catalysts under different conditions: CO + O_2 + H_2 (squares), CO + O_2 + H_2 + CO_2 (triangles), CO + O_2 + H_2 + H_2O (diamonds), CO + O_2 + H_2 + CO_2 + H_2O (circles).

X-Ray Diffraction

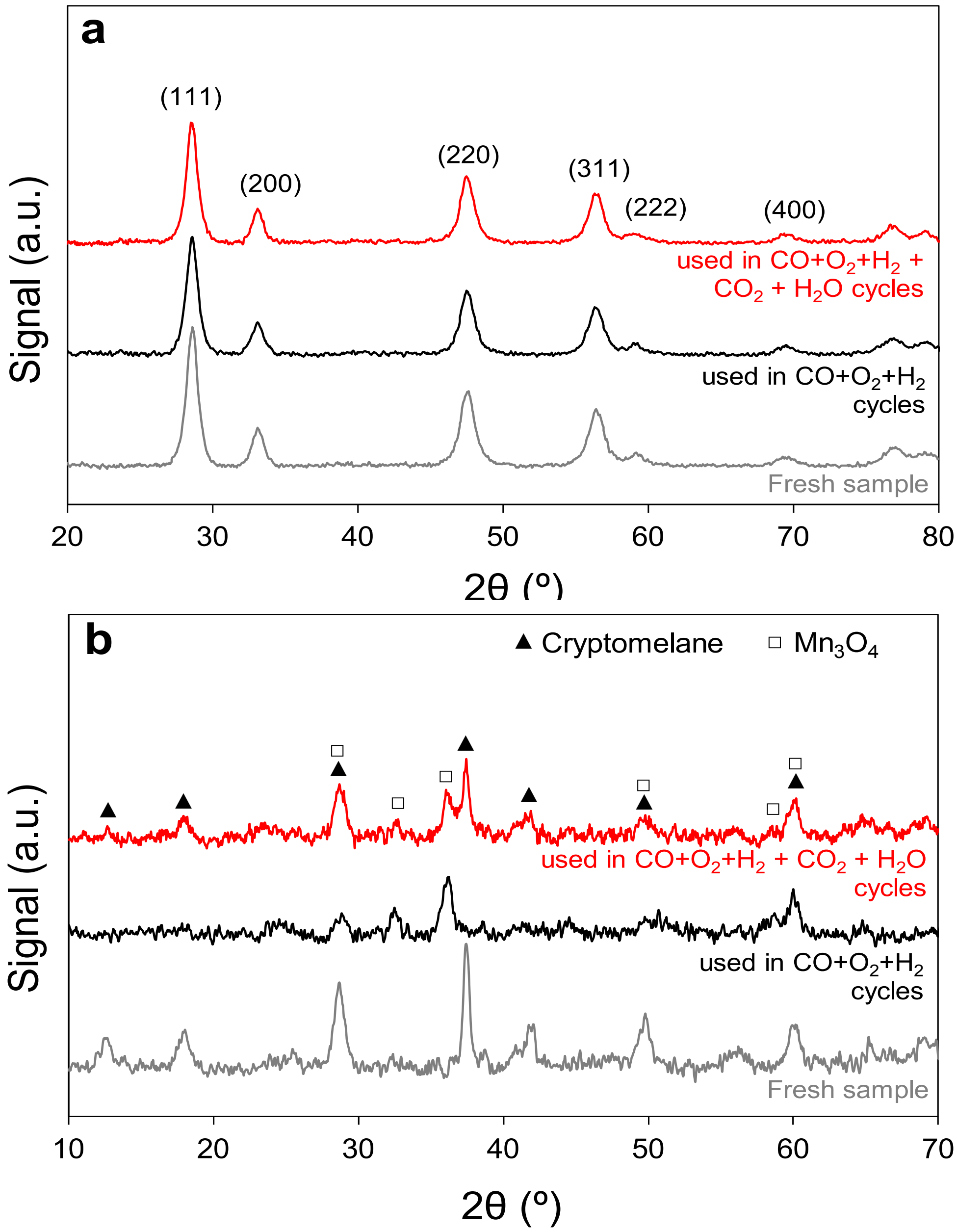

Figure S4. X-ray diffractograms for (a) CuO/CeO_2 and (b) CuO/Cryptomelane fresh and used catalysts.

Table S2. Crystallographic features of CuO/CeO_2 samples fresh and used in different conditions. Scherrer calculations and lattice constant are referred to (111) peak from cubic fluorite structure.

Sample	CeO_2 lattice constant (nm)	Crystal size Scherrer (nm)	Crystal size WH (nm)
fresh	0.539	10	14
Used in $CO+O_2+H_2$ conditions	0.540	9	15
Used in $CO+O_2+H_2$ $+CO_2+H_2O$ conditions	0.541	10	14

Table S2 displays the crystallite sizes calculated by means of Debye-Scherrer equation[1–3] and Williamson-Hall (WH),[4,5] being the lattice parameter tightly in correlation with the characteristic CeO_2 cell unit (*i.e.;* 0.5411 nm).[6]

Table S3. Crystallographic features of CuO/Cryptomelane samples fresh and used in different conditions. % α-MnO_2 calculated by peak intensity ratios

Sample	% α-MnO_2	Crystallite size Scherrer (nm)	"*a*" (nm)	"*c*" (nm)
fresh	100	17	0.981	0.286
Used in $CO+O_2+H_2$ conditions	0	11	0.575	0.932
Used in $CO+O_2+H_2$ $+CO_2+H_2O$ conditions	60	19	0.983	0.287

Scherrer calculations are referred to (211) peak from tetragonal phase centred at 37.4º.

Table S3 shows the calculated crystallite size using Scherrer equation for the (211) peak and cell parameters (*a, c*) according to cryptomelane tetragonal unit cell for the fresh and used samples. Both parameters correlate well with reported values of the cryptomelane hollandite structure (α-MnO_2), being $a = b = 0.987$ nm; and $c = 0.287$ nm.[7] In the case of CuO/Cryptomelane used sample in $CO + O_2 + H_2$ conditions, the crystalline parameters for tetragonal Mn_3O_4, the lone phase observed were calculated as $a = b = 0.575$ nm; and $c = 0.932$ nm, in close agreement with the reported JCPDS value (*i.e., a* = *b* = 0.576 nm; *c* = 0.944 nm).[8,9]

Temperature Programmed Reduction with H_2 (H_2-TPR)

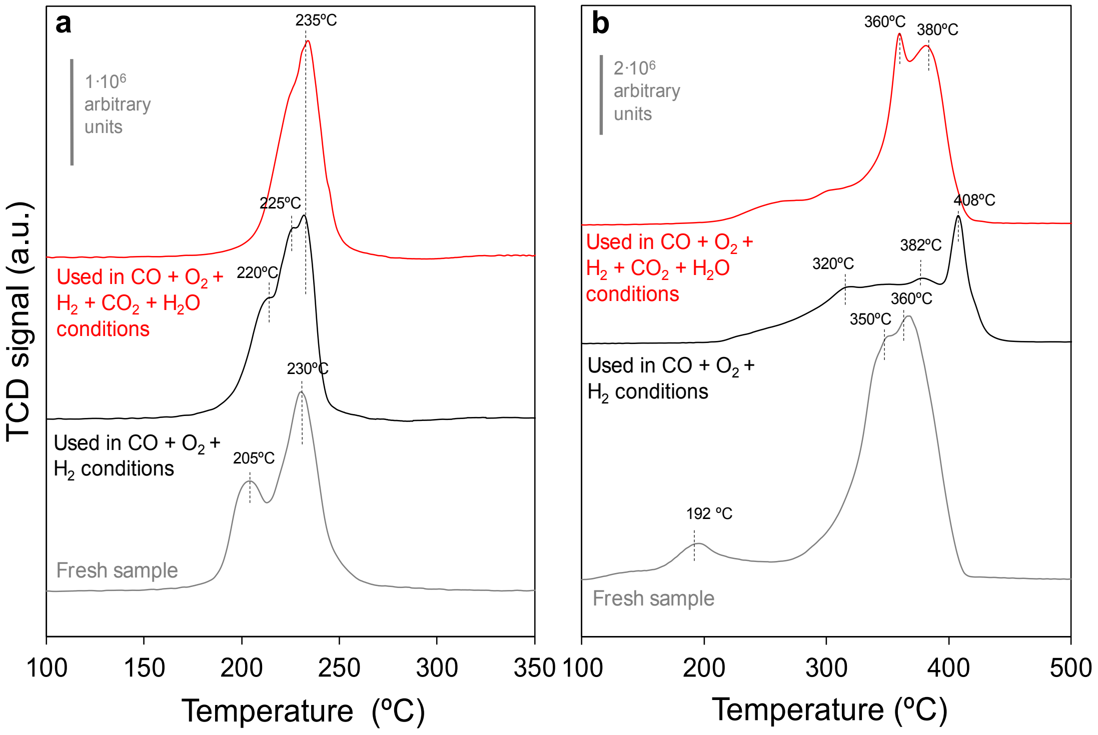

Figure S5. H_2-TPR profiles for (a) CuO/CeO_2 and (b) CuO/Cryptomelane fresh and used catalysts.

Isotopic $^{36}O_2$ Pulse Experiments

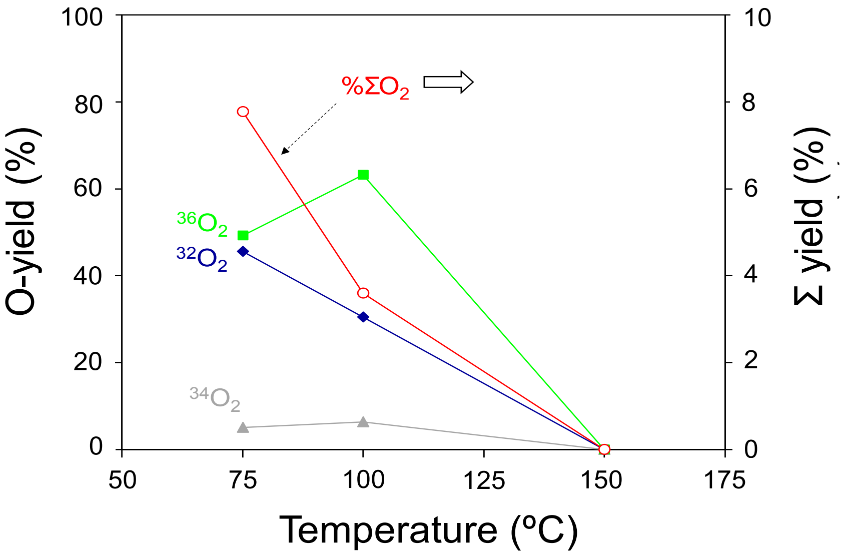

Figure S6. MS signals after $^{36}O_2$ pulses in CO-PROX conditions with CuO/Cryptomelane catalyst at 75, 100 and 150 ºC. (Solid symbols): overall O_2 released species in the outlet flow; (open symbols): isotopic distribution among H_2O and CO_2 species.

REFERENCES

(1) Jamshidijam, M.; Mangalaraja, R. V.; Akbari-Fakhrabadi, A.; Ananthakumar, S.; Chan, S. H. Effect of rare earth dopants on structural characteristics of nanoceria synthesized by combustion method. *Powder Technol.* **2014,** *253,* 304–310. https://doi.org/10.1016/j.powtec.2013.10.032.

(2) Scherrer, P. Bestimmung der Größe und der inneren Struktur von Kolloidteilchen mittels Röntgenstrahlen. *Nachr. Ges. Wiss. Goettingen, Math.-Phys. Kl.* **1918,** *1918,* 98–100. https://doi.org/10.1007/978-3-662-33915-2_7.

(3) Burton, A. W.; Ong, K.; Rea, T.; Chan, I. Y. On the estimation of average crystallite size of zeolites from the Scherrer equation: A critical evaluation of its application to zeolites with one-dimensional pore systems. *Microporous Mesoporous Mater.* **2009,** *117,* 75–90. https://doi.org/10.1016/j.micromeso.2008.06.010.

(4) Williamson, K.; Hall, W. H. X-ray line broadening from filed aluminium and wolfram. *Acta Metall.* **1953,** *1,* 22–31. https://doi.org/10.1016/0001-6160(53)90006-6.

(5) Bueno-Ferrer, C.; Parres-Esclapez, S.; Lozano-Castelló, D.; Bueno-López, A. Relationship between surface area and crystal size of pure and doped cerium oxides. *J. Rare Earths* **2010,** *28,* 647–653. https://doi.org/10.1016/S1002-0721(09)60172-1.

(6) A. Trovarelli, Catalysis by Ceria and Related Materials, Catalytic Science Series: Volume 2; Imperial College Press, 2002; pp 528. https://doi.org/10.1142/p249.

(7) Vicat, J.; Fanchon, E.; Strobel, P.; Tran-Qui, D. The structure of $K_{1.33}Mn_8O_{16}$ and cation ordering in hollandite-type structures. *Acta Crystallogr., Sect. B* **1986,** *42,* 162–167. https://doi.org/10.1107/S0108768186098415.

(8) Ezhil-Raj, A. M.; Victoria, S. G.; Jothy, V. B.; Ravidhas, C.; Wollschläger, J.; Suendorf, M.; M. Neumann, M.; Jayachandran. M.; Sanjeeviraja, C. XRD and XPS characterization of mixed valence Mn_3O_4 hausmannite thin films prepared by chemical spray pyrolysis technique. *Appl. Surf. Sci.* **2010,** *256,* 2920–2926. https://doi.org/10.1016/j.apsusc.2009.11.051.

(9) Jarosch, D. Crystal structure refinement and reflectance measurements of hausmannite, Mn_3O_4, Mineralogy and Petrology. *Mineral. Petrol.* **1987,** *37,* 15–23. https://doi.org/10.1007/BF01163155.
